# Supplementary figures and images for: Outcomes of posterior lamellar tarsal rotation vs bilamellar tarsal rotation for trachomatous trichiasis
Source: PLoS Negl Trop Dis. 2025 Jul 30;19(7):e0013152. doi: 10.1371/journal.pntd.0013152 (PMC12331057; doi:10.1371/journal.pntd.0013152)

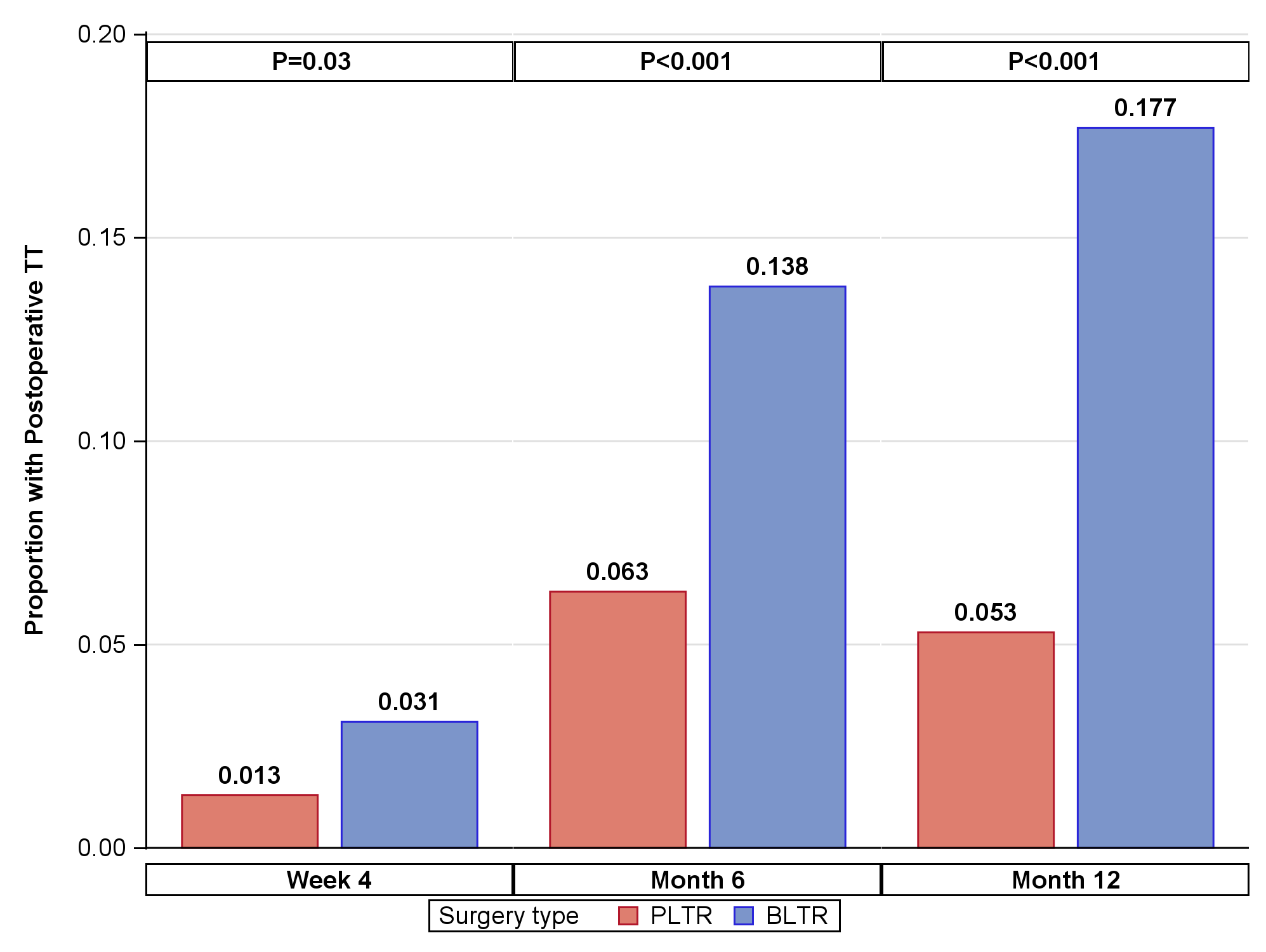

Supplement: S1 Fig — (TIFF) [file pntd.0013152.s001.tiff]
