# Supplementary material for: Outcomes of posterior lamellar tarsal rotation vs bilamellar tarsal rotation for trachomatous trichiasis
Source: PLoS Negl Trop Dis. 2025 Jul 30;19(7):e0013152. doi: 10.1371/journal.pntd.0013152 (PMC12331057; doi:10.1371/journal.pntd.0013152)
Supplement: S1 Table — (DOCX) [file pntd.0013152.s002.docx]

**S1 Table.** Eligibility Criteria for the FLuorometholone as Adjunctive MEdical Therapy for Trachomatous Trichiasis Surgery (FLAME) Trial, from whence the first 1141 consecutive participants enrolled were studied in this analysis.

**Inclusion Criteria:**

- Age 15 years or more (corresponding to field site practice patterns for surgery under local anesthesia at participating field sites);
- A decision to operate one or both upper eyelids for trachomatous trichiasis (TT) already has been made;
- Informed consent (and child assent for ages 15-17) to participate; and
- Collection of baseline information prior to randomization.

**Exclusion criteria:**

- Contraindication to study treatment or programmatic medical treatments such as azithromycin (relatively contraindicated in pregnancy);
- Elevated intraocular pressure≥22 mmHg;
- A known severe / serious ocular pathology or medical condition which may preclude study completion or increase the risk of harm in the study;
- Known or foreseen indication for systemic or ophthalmic corticosteroid therapy is indicated;
- Significant disease which might put a study participant at risk of harm if participating in the study; and
- Postoperative TT (prior TT surgery with TT present) in all eyes with TT.
