# Supplementary material for: Outcomes of posterior lamellar tarsal rotation vs bilamellar tarsal rotation for trachomatous trichiasis
Source: PLoS Negl Trop Dis. 2025 Jul 30;19(7):e0013152. doi: 10.1371/journal.pntd.0013152 (PMC12331057; doi:10.1371/journal.pntd.0013152)
Supplement: S2 Table — (DOCX) [file pntd.0013152.s003.docx]

**S2 Table.** Longitudinal Analysis for Crude and Adjusted Relative Odds for Postoperative Trachomatous Trichiasis (PTT) by Type of Surgery (BLTR vs PLTR), Visit and Baseline Factors in the FLuorometholone as Adjunctive MEdical Therapy for TT Surgery (FLAME) Trial*

| **Characteristics of Eyes** | **Postoperative TT** | | | **Crude** | | **Adjusted** | |
| --- | --- | --- | --- | --- | --- | --- | --- |
|  | **Study Eye visit, E=4570** | **No (%)** | **Yes (%)** | **Odds Ratio** | **p** | **Odds Ratio** | **p** |
|  |  |  |  | **(95% CI)** |  | **(95% CI)** |  |
| Time after randomization of follow-up visit | | | | | | | |
| Week 4 | 1529 | 1497 (97.9%) | 32 (2.1%) | Ref | <.0001 | Ref | <.0001 |
| Month 6 | 1508 | 1362 (90.3%) | 146 (9.7%) | 5.01  (3.55, 7.09) |  | 5.23  (3.71, 7.37) |  |
| Month 12 | 1533 | 1368 (89.2%) | 165 (10.8%) | 5.64  (3.95, 8.07) |  | 5.94  (4.16, 8.47) |  |
| Baseline Age in years | 4570 | Mean=47.0 (SD=15.0) | Mean=50.5 (SD=15.3) | 1.17**  (1.05, 1.31) | 0.005 | 1.19**  (1.07, 1.33) | 0.002 |
| Number of upper lid lashes touching globe/Epilating at baseline | | | | | | | |
| Zero (epilating) | 595 | 536 (90.1%) | 59 (9.9%) | Ref | 0.005 | Ref | 0.08 |
| 1-5 | 2849 | 2676 (93.9%) | 173 (6.1%) | 0.58  (0.34, 1.00) |  | 0.56  (0.32, 0.97) |  |
| 6 or more | 1126 | 1015 (90.1%) | 111 (9.9%) | 0.99  (0.55, 1.78) |  | 0.81  (0.46, 1.43) |  |
| Severity of entropion at baseline | | | | | |  | |
| None | 592 | 557 (94.1%) | 35 (5.9%) | Ref | 0.03 | Ref | 0.03 |
| Mild | 1664 | 1551 (93.2%) | 113 (6.8%) | 1.16  (0.68, 1.98) |  | 1.61  (0.92, 2.83) |  |
| Moderate | 993 | 935 (94.2%) | 58 (5.8%) | 0.99  (0.56, 1.75) |  | 1.27  (0.71, 2.28) |  |
| Severe | 1006 | 909 (90.4%) | 97 (9.6%) | 1.72  (1.01, 2.93) |  | 2.27  (1.27, 4.07) |  |
| Total | 315 | 275 (87.3%) | 40 (12.7%) | 2.36 (1.11, 5.02) |  | 3.13 (1.39, 7.03) |  |
| Upper eyelid trachomatous scarring at baseline, % | | | | | | | |
| S0: None | 407 | 374 (91.9%) | 33 (8.1%) | Ref | 0.80 | Ref | 0.80 |
| S1: Mild | 360 | 328 (91.1%) | 32 (8.9%) | 1.11 (0.56, 2.18) |  | 0.96 (0.47, 1.93) |  |
| S2: Moderate | 1856 | 1725 (92.9%) | 131 (7.1%) | 0.86 (0.50, 1.46) |  | 0.84 (0.48, 1.49) |  |
| S3: Severe | 1947 | 1800 (92.4%) | 147 (7.6%) | 0.92 (0.53, 1.62) |  | 0.99 (0.50, 1.93) |  |
| Type of TT Surgery | | | | | | | |
| BLTR | 2027 | 1793 (88.5%) | 234 (11.5%) | Ref |  | Ref | <.0001 |
| PLTR | 2543 | 2434 (95.7%) | 109 (4.3%) | 0.34 (0.23, 0.48) | <.0001 | 0.27 (0.18, 0.41) |  |
| *BLTR=bilamellar tarsal rotation; PLTR=posterior lamellar tarsal rotation; TT=trachomatous trichiasis; E=number of eyes; CI=confidence interval; Ref=reference group; SD=standard deviation; PTT refers to incidence of postoperative TT during the 12 month follow-up period.  **Odds ratios for age are counted for every ten years of increased age. | | | | | | | |
