# Supplementary material for: Outcomes of posterior lamellar tarsal rotation vs bilamellar tarsal rotation for trachomatous trichiasis
Source: PLoS Negl Trop Dis. 2025 Jul 30;19(7):e0013152. doi: 10.1371/journal.pntd.0013152 (PMC12331057; doi:10.1371/journal.pntd.0013152)
Supplement: S1 File — FLAME Research Group Members, Data and Safety Monitoring Committee Members, and National Eye Institute Program Officers, as of January 29, 2025. (DOCX) [file pntd.0013152.s005.docx]

**Credit Roster:**

**FLuorometholone as Adjunctive MEdical Therapy for TT Surgery (FLAME) Trial**

1. **RESEARCH GROUP:**

**Chair’s Office:** Massachusetts Eye and Ear/Harvard Medical School, Boston, MA,

USA

**John H. Kempen**, MD, MPH, MHS, PhD: Study Chair/Study “Sponsor”/

Overall PI

**Ahlam Awad Mohammed**, MPH, Senior Project Coordinator

**John Caccavielo**, Study Coordinator

**Shawn E. Doherty**, Budget Manager

**Geertje A. Gorfe**, MBA, Administrative Coordinator

**Anya Kamber**, Budget Manager

**Kristen Kirk-Paladino**, Senior Budget Manager and Signatory Official

**Tony Succar**, PhD, MScMed (OphthSc), Study Coordinator

**Hilkiah Kinfemichael Suga**, MBA, Post-doctoral Fellow

**Vijeeta Tadla**, PhD, PMP, CAPM, Study Coordinator

Vice-Chair: London School of Hygiene and Tropical Medicine, London, UK

**Matthew J. Burton**, PhD MA MBBCh DTM&H MRCP FRCOphth FMedSci: Study Vice-Chair

Health Economist: Johns Hopkins Carey Business School, Baltimore, MD

**K. Davina Frick**, PhD, MA

**Data Center (DC):** University of Pennsylvania Perelman School of Medicine,

Philadelphia, PA, USA

**Gui-shuang Ying**, PhD, Data Center Director/Principal Investigator for DC

**Maureen G. Maguire**, PhD, Co-investigator

**Vatinee Bunya**, MD, MSCE, Co-investigator

**Kathy McWilliams**, CCRP, Project Manager

**Erin Prachar,** Project Manager

**Yineng Chen**, MS, Biostatistician

**Fangming Jin,** MS, Biostatistician

**Judy Zhu**, Biostatistician Intern

**Patrick Augello**, Biostatistician Intern

**Susan Ryan**, Financial Administrator

**Janice Ashton**, Financial Administrator

**Claressa Whearry**, Administrative Coordinator

**Christopher Helker**, Data Management Director

**James Dattilo**, Data Manager

Investigational Drug Service:

**Vivian Leung**, PharmD, BCOP, Assistant Director

**Benedicta Asamoah**, PharmD, Clinical Pharmacy Specialist

**Claudia Mendez-Garcia**, PhD, Laboratory Manager

**Field Coordinating Center (FCC)**: Berhan Public Health & Eye Care Consultancy,

Addis Ababa and Jimma, Ethiopia

**Aida Abashawl,** MD, MPH, MS, Field Coordinating Center Director/PI

**Ahlam Awad Mohammed**, MPH, Senior Project Coordinator

**Berhanu Tulu**, MSc, Field Coordinator

**Genemo Abdela**, MSc, Field Study Supervisor

**Anbesie Wondimu**, MA, Study Nurse

**Tofik Biya**, Study Nurse

**Adugna Hora**, Study Nurse

**Kebede Sheberu**, Study Nurse

**Abebe Taku**, MSc, Study Nurse

**Daba Kumera**, Study Nurse

**Dejene Bikila**, Study Nurse

**Saliya Teyib**, Back-up Study Nurse

**Yemisirach Zenebe**, Study Data Recorder

**Gelana Kenea**, Study Data Recorder

**Kanenus Kebeta**, MSc, Study Data Recorder

**Mohamad Jemal**, Study Data Recorder

**Abdeta Tesfaye**, Study Data Recorder

**Shiferaw Asefa,** Study Data Recorder

**Birihanu Shewareged**, Field Pharmacist

**Aemero Abataneh**, MD, Safety Officer

**Leul Garaw**, Data and IT Support

**Menilik Alemayehu**, MPH, Internal Monitor

**Yohannes Sitotaw**, MSc, Internal Monitor

**Hilina Assiged,** Study Administrative Assistant in Addis Ababa

**Askual Musse**, Study Administrator Assistant in Jimma

**Yanbel Abebe**, Study Logistics Coordinator

**Senait Wassihun**, Berhan PHECC Office Administrator

**Netsanet Solomon**, Berhan PHECC Financial Administrator

**Surgical Team**: The Fred Hollows Foundation, Melbourne, Australia; Addis

Ababa and Jimma, Ethiopia

**Sarity Dodson**, PhD, Surgical Team Director/PI

**Wondu Alemayehu**, MD, MPH, Co-investigator

**Alemu Gemechu**, MPH, MBA, Co-investigator

**Alemayehu Megersa,** MPH, TT surgery program

**Tolossa Cheru,** MPH, Project Coordinator

**Gadisa Mohammad,** MSc, Zonal Program Advisor

**Oromia Regional Health Bureau**:

**Dereje Adugna Kumsa**, MPH, Co-investigator

**Hirpa Miecha**, MPH, Former NTD Lead and Co-investigator

**Jimma Zone health office**

**Fuad Sabit**, MPH, Head of Jimma Zone Health Office

**Dereje Eshete**, MPH, Jimma Zone NTD Focal Person

**Jimma zone TT surgeons who operated on FLAME trial study participants**:

**Abduletif Abajihad**

**Ahimed Mohammed**

**Ahimed Reshid**

**Alemayehu Birhanu**

**Anbessa Tesfaye**

**Awal Abajihad**

**Bulti Aga**

**Daniel Terefe**

**Diriba Wakwaya**

**Diribe Tariku**

**Endashaw Beshir**

**Eyuel Alemnah**

**Fuad Degefu**

**Gadisa Tarfesa**

**Gurmesa Wakuma**

**Huzefi Rijal**

**Jihad Reshid**

**Kinfe Bazabih**

**Malkamu Derege**

**Muktar Abanega**

**Reis Abadembel**

**Samuel Gazu**

**Semira Nasir**

**Shumet Hassen**

**Sileshi Gudina**

**Tesema Bekele**

**Tsagaye Koricha**

**Wonde Tekalign**

**Wondimagegn Hailu**

**Yilikal Mezgebu**

1. **DATA AND SAFETY MONITORING COMMITTEE (DSMC)**:

**David C. Musch**, PhD, MPH (DSMC Chair), University of Michigan School of

Medicine, Ann Arbor, MI, USA

**Jeremy D. Keenan**, MD (DSMC Safety Officer), Francis I. Proctor Founda-

tion, University of California, San Francisco, San Francisco, CA USA

**Tianjing Li**, MD, MHS, PhD (Member), University of Colorado School of

Medicine, Denver, CO, USA

**Paul Ndebele**, PhD (Member), Department of Global Health, George

Washington University Milken Institute School of Public Health,

Washington, DC, USA

**Andrea B. Troxel**, ScD (Member), New York University School of Medicine,

New York, NY USA

**Meraf A. Wolle**, MD, MPH (Former Member), Kaiser Permanente, Largo

MD USA

1. **NATIONAL EYE INSTITUTE, NATIONAL INSTITUTES OF HEALTH**: Bethesda, MD, USA.

**Sangeeta Bhargava**, PhD, Program Officer

**Jimmy Le**, ScD, Program Officer
