# Supplementary material for: Outcomes of posterior lamellar tarsal rotation vs bilamellar tarsal rotation for trachomatous trichiasis
Source: PLoS Negl Trop Dis. 2025 Jul 30;19(7):e0013152. doi: 10.1371/journal.pntd.0013152 (PMC12331057; doi:10.1371/journal.pntd.0013152)
Supplement: S1 Data — (DOCX) [file pntd.0013152.s006.docx]

**Source Data Instruction of Tables and Figures**

| **Table Name** | **Source Data in Excel sheet** | **Additional Information** |
| --- | --- | --- |
| Table 1 | Patient-level Data |  |
| Table 2 | Eye-level Data |  |
| Table 3 | Surgeon Data |  |
| Table 4 | Eye-level Data |  |
| Table 5 | Eye-level Data |  |
| Table 6 | Eye-level Data |  |
| S2 Table | Eye-level Data |  |
| Fig 1 | Survival Analysis Data | Follow-up time (days): when a patient finished the month 12 follow-up visit and didn’t develop postoperative trachomatous trichiasis at any follow-up visit, follow-up time was assigned 380 days for plotting purpose. |
