# Supplementary material for: Outcomes of posterior lamellar tarsal rotation vs bilamellar tarsal rotation for trachomatous trichiasis
Source: PLoS Negl Trop Dis. 2025 Jul 30;19(7):e0013152. doi: 10.1371/journal.pntd.0013152 (PMC12331057; doi:10.1371/journal.pntd.0013152)
Supplement: Attachment — Submitted filename: PNTD-D-24-01260_Ghasemian_20241029.docx [file pntd.0013152.s007.docx]

**Outcomes of Posterior Lamellar Tarsal Rotation vs Bilamellar Tarsal Rotation for Trachomatous Trichiasis**

**Abstract**

“Findings: Most baseline TT severity markers were statistically significantly worse in the PLTR (855 eyes) than the BLTR (678 eyes) group and PLTR surgeons were less experienced than BLTR surgeons.”

To clarify the statistical significance of the findings, please provide the results of the statistical tests (e.g., p-values) to support the claim that 'TT severity markers were statistically significantly worse in the PLTR group.' Alternatively, if these findings are based on several observations and cannot be all presented in the abstract, the authors may consider rephrasing to avoid implying statistical significance.

**Research in context**

Line 145 _ please provide the full term the first time 'PTT' is mentioned in this section to ensure clarity for readers.

In addition, I noticed that the abbreviation 'PTT' was defined and used in line 169 of the introduction, however, the full term was used repeatedly in the manuscript (lines 194, 207, 234, …). Could you please make sure that you either use the full term or the abbreviation throughout the paper to ensure clarity for readers?

**Introduction**

Line 151-153 _ please add your references.

Line 164-165 _ the statement, “TT is the primary pathway to trachoma blindness via trichiatic lashes irritating the ocular surface, causing corneal scarring and opacification,” could be misleading. For clarity, consider the following excerpt from Gupta *et al*. 2022 (PMID: 36574461):

Trachoma starts as a chronic keratoconjunctivitis caused by repeated episodes of infection with serovars A, B, Ba and C of Chlamydia trachomatis. Recurrent episodes of conjunctivitis result in conjunctival scarring, which can eventually lead to misdirection of eyelashes and trichiasis. Trachomatous trichiasis (TT) is the major risk factor for trachomatous corneal opacification, so treating trichiasis is central to preventing visual loss and blindness due to trachoma.

Line 179 _ in the final paragraph of the introduction, while the authors emphasize the significance of comparing PLTR and BLTR techniques, they introduce, for the first time, that this study was conducted “during a randomized clinical trial evaluating a separate issue—whether ancillary treatment with fluorometholone 0.1% contributes to better outcomes of TT surgery.” This statement may be confusing for readers, particularly because it does not clarify the potential implications of the ongoing clinical trial on postoperative outcomes in TT. I recommend to slightly expand on the current description of the ongoing trial, and better justify how the study population for this analysis was selected from the ongoing trial.

**Methodology**

Major comments

The current methodology section does not provide a clear path on how the study design would lead to answer the study questions. To improve readability and alignment with the study objectives, consider organizing the methodology section into subsections, such as “Study design and participants,” “Procedures,” “Measuring primary and secondary outcome,” and “Statistical analysis.” This structure would provide a clearer and more logical flow, allowing readers to follow the methodology more easily.

Additionally, the section currently opens with an explanation of the FLAME trial (line 186), which could be confusing, as this trial appears unrelated to the specific focus of the present study. I recommend beginning the methodology section by clearly defining the study design, then mentioning, as a part of this background, that the participants were recruited within the framework of the FLAME trial. This restructuring would clarify the connection to the FLAME trial without detracting from the primary focus of your study.

The current methodology section does not include any information regarding ethical approval or consent. To ensure compliance with ethical standards please add a statement that addresses the ethical aspects of this research. This should include details on the ethical approval obtained from an appropriate institutional review board or ethics committee, as well as information on informed consent from participants.

Minor comments

Line 204-209 _ this paragraph combines two distinct topics: (1) lines 204-207, which appear to relate to study procedures, and (2) lines 207-209, which pertain to the measurement of the primary outcome. Please consider separating these sentences and placing them in the appropriate subsections when restructuring the methodology.

Line 211 _ please specify that both the inclusion and exclusion criteria are listed in Supplementary Table 1. Additionally, I recommend enhancing the design of Supplementary Table 1, as the current format could be improved for better visual appeal and readability.

**Results**

In Table 2, there is a significantly higher baseline prevalence of upper eyelid trachomatous scarring in the BLTR group compared to the PLTR group (p < 0.001). Could the observed differences in the distribution of upper eyelid trachomatous scarring be associated with an increased risk of recurrent trichiasis in the BLTR group relative to the PLTR group?

Lines 277-281 _ Please reference Table 4 when discussing both the number of lashes touching the eyes (line 278) and postoperative entropion (line 281). Additionally, as the statistical data for postoperative entropion are already provided in Table 4, this information can be removed from the text to avoid redundancy.

Figure 1. Please specify the scale used on the y-axis.

Figure 2. This figure does not present any additional information beyond what is already provided in Table 4. To improve the clarity and efficiency of the results presentation, I suggest relocating Figure 2 to the supplementary materials. Furthermore, please incorporate the statistical significance into the graph to enhance understanding and context for the findings.

Line 312 _ The link to Supplementary Table 2 appears to be broken. Additionally, this table is currently presented in the main text (line 314). Please ensure that it is relocated to the supplementary materials as intended.

Could you please clarify whether any data were collected regarding the occurrence of postoperative infections in the eyes of participants undergoing BLTR compared to those receiving PLTR?

**Discussion**

Major comments

The discussion section of this paper requires substantial improvement. Firstly, the results are not thoroughly discussed, leading to a lack of clarity and depth in understanding the outcomes of the study. Additionally, the practical implications of the findings and how they should be incorporated into future WHO programs are not adequately addressed, which is essential for the effective application of this research.

Furthermore, a better comparison of the achieved data with previous studies is needed. Contextualizing the results within the framework of prior studies would enhance the discussion and provide a clearer picture of the study's contributions to the field.

Minor comments

Line 360 and 362 _ please add the reference.

Line 385 _ I find it challenging to consider the greater experience of PLTR surgeons compared to those performing BLTR as a strength of the study.

Line 384-403 _ the limitations and strengths paragraph is currently quite lengthy. I recommend revising it to either present the information as two separate paragraphs or to shorten it for improved readability.
