# Supplementary material for: Outcomes of posterior lamellar tarsal rotation vs bilamellar tarsal rotation for trachomatous trichiasis
Source: PLoS Negl Trop Dis. 2025 Jul 30;19(7):e0013152. doi: 10.1371/journal.pntd.0013152 (PMC12331057; doi:10.1371/journal.pntd.0013152)
Supplement: Attachment — Submitted filename: Responses to Reviewer Feedback.25.0314.docx [file pntd.0013152.s009.docx]

| **Reviewer Feedback** | **Response** | **Changes to manuscript** |
| --- | --- | --- |
| **GENERAL FEEDBACK** |  |  |
| **PLOS EDITORS** |  |  |
| While this is an important paper, some significant limitations need to be addressed. | Thank you for your affirmation of the value of the paper. We address the specific issues raised below | See below |
| New clinical trial has been published, please incorporate it. | Thank you for pointing this out. We were awaiting this trial, but it was not ready at the time we were preparing this manuscript. We have carefully reviewed the new trial and incorporated its information into our manuscript. | The trial provides important discussion points regarding the interpretation of PLTR results from our paper. We have cited it extensively in the revision and with regard to policy implications. |
| Supporting Information File Legends | I interpreted this request as meaning I should add legends for the Supplemental Figure and Supplemental Tables and Credit Roster at the end of the manuscript, which has been done. It is only in the “clean” manuscript since it will not work to put it at the end with the End Note program still active | Added after the references in the manuscript text file. |
| Request to submit the data supporting the report. | The data, and a document describing which parts of the dataset support which figures and tables, have been provided | Supplemental Materials |
| Updates to several numbers in the tables without qualitative differences. | In the process of preparing the data for submission and verifying results, we discovered one observation that had been misclassified which changed several numbers in Tables 1,2,3, and 5. These have been corrected. Because only track changes are requested for the manuscript itself, we have not included tracked changes in the Tables, but can if need be. The changes did not result in any qualitative differences such as something that was no longer significant. | Tables 1,2,3, and 5 |
|  |  |  |
| **Reviewer 2:** |  |  |
| Use PTT after defining it | Thank you for pointing this out | PTT used in the places subsequent to when it was first defined in the manuscript text. Tables, Figures, and Research in Context are counted as separate sections requiring their own definition of the acronym (if it needs to be defined in each) |
|  |  |  |
| **ABSTRACT** |  |  |
| **Reviewer 2:** |  |  |
| Clarify the basis of “statistical significance” of differences between severity markers between the BLTR and PLTR groups | Indeed the comment is based on several observations which are too many to cover with the abstract word limits. So we have rephrased as suggested. | See abstract, findings, we deleted “statistically significantly”: while the differences are significant, we are unable to specify the comparisons with the available word count. |
|  |  |  |
| **RESEARCH IN CONTEXT** |  |  |
| **Reviewer 2:** |  |  |
| Define PTT the first time it is used | We decided not to use the acronym in the “Research in Context” section | “PTT” is changed to “postoperative TT” |
|  |  |  |
| **INTRODUCTION** |  |  |
| **Reviewer 2:** |  |  |
| Add references for lines 151-153 | References 1-5 are given. I am trying to economize on the number of references | Introduction, first paragraph |
| Lines 164-165: suggest using the approach of Gupta et al (PMID 36574461) | I paraphrased the material Reviewer 2 suggested as the first sentence of Introduction paragraph 3 | Introduction, paragraph 3. |
| Line 179: recommend expanding a bit on the description of the parent clinical trial. | We have expanded on the parent trial’s design in the last paragraph of the introduction and in the Methods paragraph 1. | Introduction, paragraph 4.  Methods, paragraph 1. |
|  |  |  |
| **METHODS** |  |  |
| **Reviewer 1:** | We appreciate Reviewer 1’s favorable review | No changes in response |
| **Reviewer 2:** |  |  |
| Clarify how the study design addresses the research questions. | Although the parent study is randomized clinical trial comparing the effect of active treatment with fluorometholone 0.1% twice daily for 28 days on and following TT surgery, it functions as cohort study for non-randomized variables such as PLTR vs BLTR surgery | Introduction, paragraph 4.  Methods, paragraph 1. |
| More details re: population under study | The study sponsored the TT surgery outreach program in the Jimma zone, Oromia Region, Ethiopia—a poor rural community with high trachoma burden. Persons for whom first TT surgery was planned for at least one upper eyelid were screened for eligibility and recruited to the study. Those who wished to participate were enrolled, about 90% of those screened. | Methods, Study Design subheading, paragraph 2; Methods, Participants subheading only paragraph; Supplemental Table 1. |
| Specify the ethics committee review | This study was approved at outset and approval regularly extended as required by each of the following IRBs :  Ethiopian authorities:   1) Ethiopian Food and Drug Administration; 2) National Research Ethics Review Cmte 3) Mass General Brigham IRB, the IRB for the PI and grant recipient 4) London School of Hygiene and Tropical Medicine IRB, the IRB for the study Vice-Chair.   All participants provided written informed consent. Consent materials were available in the three languages appropriate to the region, most using the Oromo language. | See the new “**Ethical Approval and Informed Consent”** section, the last section in the Methods in the revised version. |
| To improve readability and alignment with the study objectives, consider organizing the methodology section into subsections, such as “Study design and participants,” “Procedures,” “Measuring primary and secondary outcome,” and “Statistical analysis.” | Thank you, the Methods section now has the following subheadings similar to what Reviewer 2 suggested:   - **Study Design** - **Participants** - **Procedures** - **Measurement of Primary and Secondary Outcomes, and Covariates** - **Statistical Analysis** - **Ethical Approval and Informed Consent** | Throughout the Methods section. |
| The section currently opens with an explanation of the FLAME trial (line 186), which could be confusing, as this trial appears unrelated to the specific focus of the present study. I recommend beginning the methodology section by clearly defining the study design, then mentioning, as a part of this background, that the participants were recruited within the framework of the FLAME trial | See also above responses to the Introduction section. | Introduction, paragraph 4.  Methods, paragraph 1. |
| Line 204-209 _ this paragraph combines two distinct topics: (1) lines 204-207, which appear to relate to study procedures, and (2) lines 207-209, which pertain to the measurement of the primary outcome. Please consider separating these sentences and placing them in the appropriate subsections when restructuring the methodology. | This is divided into two sentences in the revised version. | First sentences in the new “Procedures” section of the Methods. |
| Line 211 _ please specify that both the inclusion and exclusion criteria are listed in Supplementary Table 1. | We have specified that eligibility criteria include both inclusion and exclusion criteria, and we briefly summarize them in the text | First sentence of the new “Participants” section of the Methods. |
| Additionally, I recommend enhancing the design of Supplementary Table 1, as the current format could be improved for better visual appeal and readability | Supplemental Table 1 has been revised, removing the lines to make it less busy and more readable | Supplemental Table 1 |
|  |  |  |
| **Reviewer 3:** |  |  |
| Provide clearer definitions regarding adverse outcomes studied (listed to right): | Eyelid contour abnormality: The distortion of the normal eye lid curvature(contour) to notching or wavey eye lid margin shape. The severity is determined by the vertical deviation of the eye lid margin from the natural contour or the extent of the horizontal eye lid margin involvement.  Eyelid notch: A dimple or groove on the upper eye lid margin the severity depends on the vertical deviation of the eye lid margin from the normal eye lid contour line. It is a subset of eyelid contour abnormality.  Overcorrection: The over rotation of the upper eyelid margin (more than 2 mm) after trachomatous trichiasis surgery.  Trachomatous scarring: The variable levels of none, mild, moderate, severe were derived from this primary variable:   - CO: No scarring on the conjunctiva - C1: Mild: fine scattered scars on the upper tarsal conjunctiva, or scars on the other parts of the conjunctiva - C2: Moderate: more severe scarring but without shortening or distortion of the upper tarsus - C3: Severe: scarring with distortion of the upper tarsus | The definitions have been added to the footnotes of Tables 2, 4, and 6. |
| Specify training of study team (standardization, monitoring for drift) | Baseline and periodic refresher trainings in outcome ascertainment were conducted for study nurses who assessed outcomes by a well-recognized trachoma expert ophthalmologist (AA). A supervising senior nurse regularly duplicated assessments on a subset of study participants to confirm the accuracy of gradings, and to forestall drift over time, which were evaluated over time using agreement statistics such as kappa. Results were favorable. | See last sentence of **Measurement of Primary and Secondary Outcomes, and Covariates,** in the **Methods** section**.** |
| Recommend assessing individual adverse events individually rather than in aggregate. | We took the approach of starting with a general comparison that adverse outcomes tended to be more common with BLTR initially and then became similar in the groups over more follow-up. Individual comparisons also are provided, both in the last paragraph of the Results section and in Table 6. | The individual comparisons are provided in the last paragraph of the Results and in Table 6. |
|  |  |  |
| **RESULTS** |  |  |
| **Reviewer 1:** |  |  |
| In Table 1, percentages exceeding 100 are shown. This needs to be corrected | We regret the error. | Fixed in Table 1. The other percentages are confirmed and were accurate. |
|  |  |  |
| **Reviewer 2:** |  |  |
| In Table 2, there is a significantly higher baseline prevalence of upper eyelid trachomatous scarring in the BLTR group compared to the PLTR group (p < 0.001). Could the observed differences in the distribution of upper eyelid trachomatous scarring be associated with an increased risk of recurrent trichiasis in the BLTR group relative to the PLTR group? | Thank you for this feedback. There actually is more severe scarring in the PLTR group. While there is more moderate scarring in the BLTR group, moderate + severe is more in the PLTR group, and the p value refers to a more severe distribution in the PLTR group. Thus, it seems unlikely the distribution of trachomatous scarring would explain the result. (We also adjust for these factors using multiple regression). | No change. |
| Lines 277-281 _ Please reference Table 4 when discussing both the number of lashes touching the eyes (line 278) and postoperative entropion (line 281). Additionally, as the statistical data for postoperative entropion are already provided in Table 4, this information can be removed from the text to avoid redundancy. | Thank you kindly. However, I prefer not to repeatedly call out the same table in three successive sentences, since I think it is adequately clear that I am continuing to discuss it. We have omitted the p values in the text, but I find it to be more informative to have the 95% CI’s. If the Editor wants me to change these, I am okay with it, but it is more my style like it is. |  |
| Figure 1. Please specify the scale used on the y-axis. | Thank you kindly, we have changed from rate to proportion which is better. | Figure 1. |
| Figure 2. This figure does not present any additional information beyond what is already provided in Table 4. To improve the clarity and efficiency of the results presentation, I suggest relocating Figure 2 to the supplementary materials. Furthermore, please incorporate the statistical significance into the graph to enhance understanding and context for the findings. | Thank you, we agree. We have changed it to Supplemental Figure 1 and have incorporated the p values at the top of each set of columns | Figure 2 is now Supplemental Figure 1. |
| Line 312 _ The link to Supplementary Table 2 appears to be broken. Additionally, this table is currently presented in the main text (line 314). Please ensure that it is relocated to the supplementary materials as intended. | Thanks, we changed the link to a placeholder to the PLOS link to the Supplement. | Results, fourth paragraph, last sentence. |
| Could you please clarify whether any data were collected regarding the occurrence of postoperative infections in the eyes of participants undergoing BLTR compared to those receiving PLTR? | Yes, there were data collected about this topic. They did not significantly or substantially differ and were very uncommon. | Results, fifth paragraph, 2^nd^ and last sentences. |
|  |  |  |
| **Reviewer 3:** |  |  |
| Table 3: Please check total versus last year surgeries for both groups, exactly the same, which doesn't make sense given the number of years of surgery reported | Thank you for catching that. Table 3 has been updated accordingly. | Table 3 |
| Table 4: By definition, the way the analyses are reported, the BLTR group would have to have a higher number of lashes, since more eyes had TT. | We agree that this is likely, and mention this point now in the text “….correspondingly lower in PLTR…” We have kept it in the Table since the number of lashes is relevant as a pathogenetic pathway. | Results paragraph 2, last sentence. |
| Table 4: would be more informative if the data about PTT severity was limited to eyes that had PLTT. | We calculated it this way, but it changes the theme of the table: from looking at the outcome by treatment group to looking at whether those who have had the outcome differ (they don’t). So we have left it as is except for adding a comparison of the counts of lashes among those with PTT in a footnote in response to the feedback. | Table 4 |
| Table 4: Consider just making 1 footnote for gee, in the column heading, instead of having it in every row. | We have done so. | Table 4. |
| Lines 299-304: This section is unclear. An eyelid can have epilation, trichiatic eyelashes, or both. So it is unclear why the comparisons were made 6 or more vs epilation, 1-5 vs epilation, etc. Typically in TT surgery trials, eyelids are assigned a severity level based on number of eyelashes touching the eye and/or amount of epilation. | We prefer to leave it with the primary data as is, there also is some uncertainty in inferring the severity of epilation. | No change. |
| Line 312 references a lancet website. Is this correct? | Thank you for pointing out the error, we have updated it to indicate where the PLOS NTD website link will go once it is available. | Second to last paragraph of Results, last sentence. |
| Supplemental table 2: Details are needed on the timing of the variables shown - are the rows baseline levels or the amount at follow up? | They are baseline values, this is now clarified | Supplemental Table 2. |
|  |  |  |
| **Conclusions** |  |  |
| **Reviewer 1**: |  |  |
| The results of this study are of considerable clinical importance and should lead to a change in the WHO recommendations for trichiasis surgery. | We appreciate Reviewer 1’s affirmation of the importance of the study | No changes |
|  |  |  |
| **Reviewer 2**: |  |  |
| The discussion section of this paper requires substantial improvement. Firstly, the results are not thoroughly discussed, leading to a lack of clarity and depth in understanding the outcomes of the study. Additionally, the practical implications of the findings and how they should be incorporated into future WHO programs are not adequately addressed, which is essential for the effective application of this research. | The Discussion has been updated taking the Reviewer’s guidance into consideration | Discussion section |
| Furthermore, a better comparison of the achieved data with previous studies is needed. Contextualizing the results within the framework of prior studies would enhance the discussion and provide a clearer picture of the study's contributions to the field. | In the Discussion, this part also has been addressed. | Discussion |
| Line 360 and 362 _ please add the reference. | References added. | Discussion, paragraph 1, sentence 4. |
| Line 385 _ I find it challenging to consider the greater experience of PLTR surgeons compared to those performing BLTR as a strength of the study. | We raised this issue, because a criticism of the RCT comparing PLTR vs BLTR by Habtamu et al (ref 7 in the manuscript) was that Habtamu’s surgeons were argued to be more experienced in PLTR than BLTR which created an alternative explanation for the difference observed in favor of PLTR. In our study the “PLTR surgeons were NOT more experienced than BLTR surgeons” as we put it in the text (emphasis on NOT added). | Discussion, paragraph 5 (strengths), first sentence: We italicized “not” to avoid confusion among readers, and added “(addressing a potential criticism regarding the trial of Habtamu et al^7^).” |
| Line 384-403 _ the limitations and strengths paragraph is currently quite lengthy. I recommend revising it to either present the information as two separate paragraphs or to shorten it for improved readability. | We have divided strengths and limitations into two paragraphs. | Discussion, paragraphs 4 and 5. |
|  |  |  |
| **Reviewer 3**: |  |  |
| Determination cannot be made with regards to whether the conclusions are supported by the data untiladditional data are provided. | Additional data are provided herein, responding to the valuable feedback of the reviewers | [See the itemized responses to the specific points] |
|  |  |  |
| **EDITORIAL AND DATA PRESENTATION MODIFICATIONS** |  |  |
|  |  |  |
| **Reviewer 1:** |  |  |
| The percentages in table 1 need to be corrected | We thank the Reviewer for picking up this typo | Fixed in Table 1. |
|  |  |  |
| **Reviewer 2:** |  |  |
| Reviewer 2’s comments on these topics were included in the other sections. | No comments in this category | No changes. |
|  |  |  |
| **Reviewer 3:** |  |  |
| There are a number of inconsistencies in terminology, typos, and potential lack of proofreading the submission – for example, line 187 "study's design study paper", line 197 "block, size 2 or 4" (comma in the wrong place), "trachoma-tous" used in all tables, line 238 "one year's follow up", abstract "PTLR" instead of "PLTR", line 166 Posterior TarsalRotation (should be Posterior Lamellar Tarsal Rotation), table 4 "percent of have", line 311 "at each follow up visits",etc. | We appreciate the Reviewer’s detailed feedback. We have carefully reviewed these specific instances and the whole manuscript and corrected accordingly. | Throughout the paper |
| Line 150-151: Consider rewording, as "primarily mediated by" suggests that there are multiple components of trachoma that cause blindness, but to this reviewer's knowledge, corneal opacity caused by TT is the necessary pathway for blindness in trachoma. | We have redone this piece in accordance with the recommendations of Reviewer 2 | Introduction, paragraph 3. |
| Line 170 what is meant by "a difference which was *qualitatively* sustained"? | We meant that that the interpretation was not changed over time but the exact numbers were not. To avoid confusion the term apparently introduced, we have deleted it. | Introduction, paragraph 3, Sentence 4. |
| Line 217 (and elsewhere) "extant" suggests something old but still surviving. Consider a different word that conveys a thriving, active surgical program? | We changed extant to ongoing | Methods, Procedures, last sentence. |
| Consider using "participants" or "subjects" instead of "patients" | We have changed instances of “patients” to “participants” | In various places through the paper. |
| It is unclear why PLTR and BLTR are referred to as "alternative" procedures. These are the 2 standard surgical procedures for TT. | “Alternative procedures” or “alternative treatments” are terms commonly used to indicate the two treatments that are being compared. We don’t think it needs to be changed. | No change. |
|  |  |  |
| **Additional Changes** |  |  |
| Additional Author | During the time since submission, the masters’ level statistician who crunched the statistics (Yineng Chen) resigned to pursue a PhD, and a large amount of statistical work had to be done in response to the above points. Therefore, I am adding Fangming Jin as an additional coauthor. She has contributed substantially to the statistical analysis now being reported (Data curation, formal analysis, methodology, visualization, and Writing-review & editing). Her contributions are being updated in the “Contributor Roles” of the submission interface.  We have submitted the required materials to add an author and it was accepted by the editorial department | Authors. |
| Clarification about absence of clinical trial materials in the submission (not a primary clinical trial report) | We do not submit any CONSORT materials since this is an observational study using the parent clinical trial’s database, not a clinical trial report. Neither do we submit the clinical trial’s protocol, lest we lose the copyright, since it will have to be submitted with the primary results report. |  |
